# Hermann Rorschach: From klecksography to psychiatry

**DOI:** 10.1590/1980-57642020dn14-010013

**Published:** 2020

**Authors:** Ricardo Vieira Teles

**Affiliations:** 1 Faculdade de Medicina, Universidade Federal de Goiás (UFG), Goiânia, GO, Brazil.

**Keywords:** Rorschach method, psychodiagnostic, Hermann Rorschach, método de Rorschach, psicodiagnóstico, Hermann Rorschach

## Abstract

Hermann Rorschach was a Swiss psychiatrist and psychoanalyst, best known for developing a projective test known as the Rorschach inkblot test, a test designed to reflect unconscious parts of the personality that project into the visual stimuli generated by the inkblots, allowing a psychodiagnosis to be established. The technique he developed has been applied since 1921 in a number of countries. Although it has long remained controversial and divided opinions, this did not prevent it from overcoming the barriers of science to have a major influence on pop culture, resulting in an undeniable legacy for the development of Psychiatry in the nineteenth century.

## RESUMO

Hermann Rorschach foi um psiquiatra e psicanalista suíço, mais conhecido por desenvolver um teste projetivo conhecido como o teste da mancha de tinta de Rorschach, um teste desenhado para refletir partes inconscientes da personalidade que se projetam nos estímulos visuais gerado pelas manchas de tinta, o que possibilitaria estabelecer um psicodiagnóstico. A técnica que ele desenvolveu tem sido aplicada desde 1921 em vários países, e embora por muito tenha permanecido polêmica e dividido opiniões, isso não a impediu de ultrapassar as barreiras da ciência para alcançando massivamente a cultura pop, resultando em um legado inegável para o desenvolvimento da Psiquiatria no século XIX.

**Figure 1:**
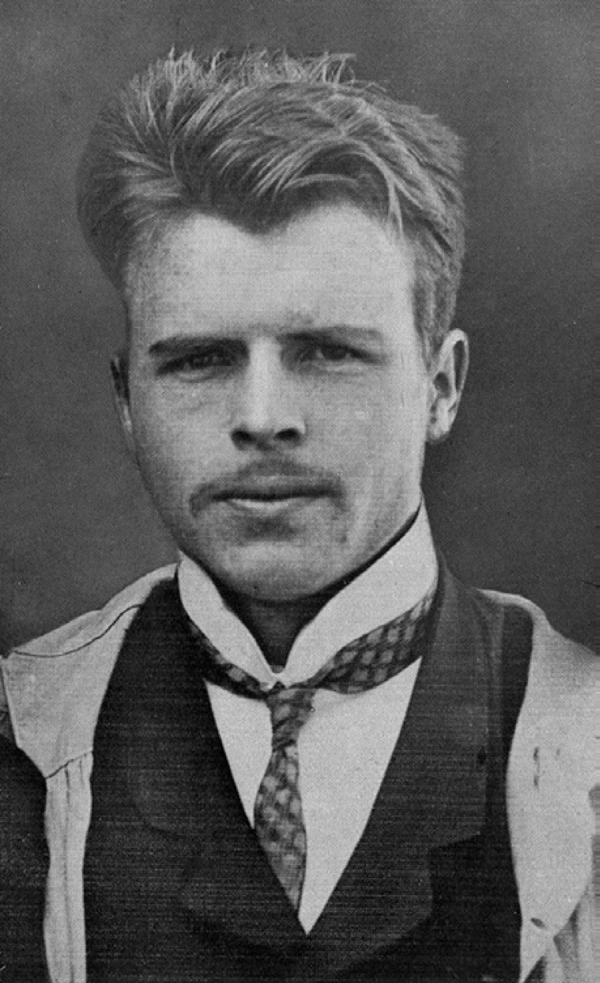
Hermann Rorschach – International Dictionary of Psychoanalysis. Alain de Mijolla (ed.). ISBN 0-02-865994-5.

Hermann Rorschach (Figure 1) was born in Zurich, Switzerland on November 8, 1884. His family was humble, with his father being a modest painter who made a living teaching art privately. Hermann showed great interest in drawing from a young age, being known by his school friends as *Klex*, or “inkblot”, since he liked Klecksography, a popular game among schoolchildren at the time, which consisted of filling a piece of paper with ink and then folding it, thus obtaining singular and fun figures. Befittingly, the basis of Klecksography was the blurry images that would later be the foundation of his Psychodiagnosis test.

He graduated in Medicine in Zurich in 1909, a period coinciding with the widespread dissemination of research on the new ideas of a then unknown psychiatrist, Sigmund Freud.^1^ He became one of the most interested listeners, as he followed discussions and communications about Freudian revolutionary innovations, in which the unconscious was no longer treated merely as a philosophical abstraction, but as the fruit of scientific inquiry.

In 1911, he began his studies and research with inkblots; yet his concern went further than the mere study of imagination and fantasy, involving the search for a method of personality investigation, situating the interpretation of inkblots in the field of perception and apperception. On November 12, 1912, he received the title of Doctor of Medicine at the University of Zurich and published the work *Reflex Hallucinations and Symbolism*, a further step towards his psychodiagnosis. Influenced by the psychoanalytic school, Rorschach, along with Otto Biswanger and other colleagues, he founded the Zurich Psychoanalysis Society.^2^

In 1914, he specialized in psychiatry at the University of Zurich. Between 1915 and 1922, Rorschach worked at Herisau Hospital as chief physician, assisted by Hans Behn Eschenburg, who would later create a parallel series of inkblots known as the Behn-Rorschach test. Symon Hens’s work in 1917 had the greatest influence on Rorschach during this period. Hens used 8 cards with stains of uncolored inks, investigating the content of the answers given by children, normal adults and psychotic patients.^3^

**Figure 2:**
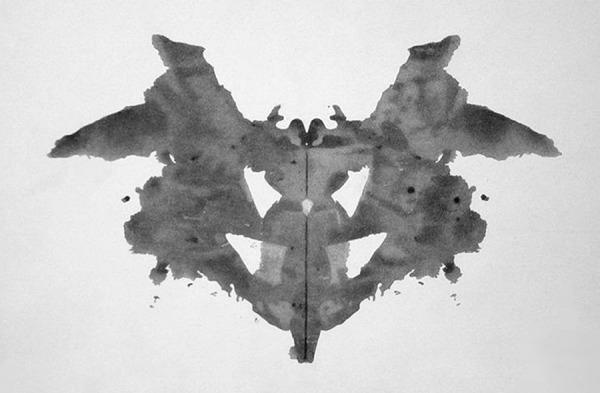
The first of the blots of the Rorschach inkblot test – Wikimedia.

In the following year (1918), Hermann Rorschach created 15 boards with two structuring elements: axis and symmetry. Some of them were black and white, some were black and red, and others were colored (Figure 2). He began to experiment with them on his patients at Herisau Hospital, as well as nurses, medical students, children, and others, comprising a sample of 288 mentally ill and 117 “normal” individuals. He observed a correlation in the responses of schizophrenic patients and theorized that mental health could be assessed by the way someone processes visual information. Rorschach then sent his test boards to a publisher to be serially printed. As request by the publisher, his boards were reduced to 10 and have been used as such ever since.^4^

In June 1921, he published the book *Psychodiagnosis*, containing the conclusions of his studies and experiments with the boards he had designed, in which Rorschach defined the foundations of the test, which he termed a projective test.^5^ He explained that his purpose was to explore people’s imaginary representations, asking them to verbally express the associations they made with the drawings shown. Prior to this, Rorschach had studied in detail the mechanisms of dreams, delirium, and hallucinations. Although always having been a follower of Freud, a Jungian influence on his concepts and language is clear.^6^ He sought the inner images and marks of civilization in the responses of his patients. His main objective was to determine whether patients were neurotic or psychotic, psychoanalytic concepts that dominated Psychiatry at the time. The basic idea is that when a person is shown a meaningless image, such as an inkblot, their mind will work hard to give meaning to this stimulus, and this attribution of meaning indicates the individual’s mental condition.

The designing of the boards, their application to patients and “normal” subjects, the writing of the book, and its difficult publication in June 1921 took place in little over three years.^7^ The following year, Rorschach died suddenly at the age of 37 from acute peritonitis due to appendicitis, shortly after publication of his work. His premature death cut short studies on the Psychodiagnostic technique.

The Rorschach Method remained restricted to a small circle of friends and followers in Switzerland. About ten years after his death, *Psychodiagnosis* began to expand and be effectively recognized in Europe and the United States. In 1939, the Rorschach Institute was created, and four years later the 1st Rorschach Congress was held. In 1949, the Rorschach International Society was founded. Few devices in the world of Psychology have penetrated popular culture as strongly as Hermann Rorschach’s famous inkblot test, which still divides psychologists from different countries regarding its questionable scientific value, mainly due to the lack of internal and external validation of the test. However, a large 2013 study published by the American Psychological Association found it more effective than previously believed for diagnosing mental illness.^8^ Today, the test is still widely used and its importance as a projective technique is scientifically recognized, constituting a unique pictorial psychological assessment.

In addition, Art honored Rorschach constantly in many ways. For example, pop artist Andy Warhol painted his own series of 38 “Rorschach’s inkblots” in 1984. Perhaps the most famous reference, Alan Moore, created the Rorschach character in the 1986 Watchmen graphic novel, an antihero who wears a mask with an inkblot and asks his victims what they see. Therefore, the influence of Rorschach’s work on popular culture is undeniable, and its contribution to the construction of knowledge in psychiatry should be recognized.^9^
